# GluCl.Cre^ON^ enables selective inhibition of molecularly defined pain circuits

**DOI:** 10.1097/j.pain.0000000000002976

**Published:** 2023-06-27

**Authors:** Steven J. Middleton, Huimin Hu, Jimena Perez-Sanchez, Sana Zuberi, Joseph McGrath Williams, Greg A. Weir, David L. Bennett

**Affiliations:** a Nuffield Department of Clinical Neurosciences, University of Oxford, Oxford, United Kingdom; b School of Psychology and Neuroscience, College of Medical, Veterinary and Life Sciences, University of Glasgow, Glasgow, United Kingdom

**Keywords:** Chemogenetics, Cre/lox, Silencing, Nociception, Pain circuits

## Abstract

Insight into nociceptive circuits will ultimately build our understanding of pain processing and aid the development of analgesic strategies. Neural circuit analysis has been advanced greatly by the development of optogenetic and chemogenetic tools, which have allowed function to be ascribed to discrete neuronal populations. Neurons of the dorsal root ganglion, which include nociceptors, have proved challenging targets for chemogenetic manipulation given specific confounds with commonly used DREADD technology. We have developed a cre/lox dependant version of the engineered glutamate-gated chloride channel (GluCl) to restrict and direct its expression to molecularly defined neuronal populations. We have generated GluCl.Cre^ON^ that selectively renders neurons expressing cre-recombinase susceptible to agonist-induced silencing. We have functionally validated our tool in multiple systems in vitro, and subsequently generated viral vectors and tested its applicability in vivo. Using Nav1.8^Cre^ mice to restrict AAV-GluCl.Cre^ON^ to nociceptors, we demonstrate effective silencing of electrical activity in vivo *and* concomitant hyposensitivity to noxious thermal and noxious mechanical pain, whereas light touch and motor function remained intact. We also demonstrated that our strategy can effectively silence inflammatory-like pain in a chemical pain model. Collectively, we have generated a novel tool that can be used to selectively silence defined neuronal circuits in vitro and in vivo. We believe that this addition to the chemogenetic tool box will facilitate further understanding of pain circuits and guide future therapeutic development.

## Abstract 2

Supplemental Digital Content is Available in the Text.

Middleton et al., have developed a novel means to selectively suppress the activity of molecularly defined populations of neurons and neural circuits.

## 1. Introduction

Noxious sensory signals transmit through nociceptive circuits originating at the sensory end organ, along the sensory neuraxis to the brain, where the perception of pain is generated. As initial transducers, nociceptors are a critical part of this circuit, and are fundamental for nociception and pain.^16,28^ Sensory neurons are highly heterogeneous and include nociceptors, pruriceptors, thermoceptors, and low threshold mechanoreceptors, a feature becoming increasingly apparent due to cell sequencing studies conducted across species.^23,39,43,46,51^ As such, there is a need to investigate the contributions of different nociceptor subpopulations to the different components of pain-related behaviours.

One approach to define nociceptor function is to activate or silence molecularly defined subtypes while assessing the consequence to sensory behaviour. Many such studies have relied on ablation of genetically defined populations, which is irreversible and may also be complicated by the inflammatory response to cell death.^1,3,7,14,27,31^ Alternatively, for controlled, fast, and spatiotemporal manipulation of neurons, optogenetic tools are well suited.^10,12,13,20,48^ However, chemogenetic tools offer the ability to reversibly activate or inactivate neurons for hours to days, which is advantageous for the study of pain and nociceptive circuits given the role played by long-term aberrant electrical activity in shaping maladaptive plasticity in the somatosensory nervous system.

Chemogenetic approaches require the expression of an engineered receptor or ion channel that is exclusively activated by an exogenous agonist. The most widely used chemogenetic tools used in neuroscience are the designer receptors exclusively activated by designer drugs (DREADDs).^4,34^ Although DREADDs have been used in the pain field to inactivate large populations of nociceptors,^20,21,30,35^ studies have raised issues with silencing efficacy,^35^ agonist conversion,^19^ and agonist-independent changes in endogenous currents and G-protein coupled receptor function.^35^

Since the discovery and application of DREADDs, others have created novel chemogenetic tools to modulate neuronal activity.^42^ These include the ivermectin (IVM) sensitive, engineered glutamate-gated chloride channel (GluCl).^24,41,49^ GluCl has been modified so that it is no longer sensitive to glutamate but highly sensitive to low doses of the agonist IVM (which is approved for human use). When activated, this channel facilitates a chloride conductance. GluCl was further optimised and used as means of silencing rodent sensory neurons and human-induced pluripotent stem cell–derived nociceptors.^49^ Activation of GluCl expressed in a nonbiased manner in mouse dorsal root ganglion (DRG) neurons leads to hyposensitivity to sensory stimuli and recovery of neuropathic pain–related hypersensitivity after nerve injury.^49^

The aim of this study is to expand the chemogenetic toolbox by generating novel GluCl constructs that are Cre-recombinase dependent (termed GluClαβ.Cre^ON^). This new tool aims to facilitate the specific targeting of molecularly defined neuronal populations using the Cre/Lox system, which can be used by the pain field and the wider neuroscience community. We have functionally validated GluClαβ.Cre^ON^ in vitro. When expressed in Nav1.8^Cre^ positive sensory neurons (a broad marker of nociceptors) in vivo, we demonstrated selective silencing of mechanical, thermal, and chemical pain-related behaviour.

## 2. Methods

### 2.1. Animals

All mice were group-housed in individually ventilated cages with free access to food and water, in humidity and temperature controlled rooms with a 12 hours light-dark cycle, in a pathogen-free facility. All animal procedures adhered to the UK Home Office (Scientific Procedures) Act (1986) and were performed under a UK Home Office Project Licence. All animal experiments were performed in accordance with the University of Oxford Policy on the Use of Animals in Scientific Research. The work within this study also conforms to the ARRIVE guidelines.^22^ All experiments were performed on adult male and female mice. No animals were excluded from this study.

The Nav1.8^Cre^ line was a gift from John Wood (UCL) and was previous generated and described.^32^ For histological studies, Nav1.8^Cre^ mice were bred with the Cre-dependent reporter line Ai14 (tdTomato; JAX). C57BL6 wild-type mice were sourced from biomedical services breeding unit at the University of Oxford.

### 2.2. Molecular cloning and viral production

Generation of GluClα.Cre^ON^ (Cerulean tagged) and GluClβ.Cre^ON^ (yellow fluorescent protein [YFP] tagged): The same procedure was performed for GluClα and GluClβ. The sequence from optimised GluClv2.0^49^ was polymerase chain reaction (PCR) amplified using primers designed with over hanging restrictions sites:

GluClα-NheI forward 5′-CTAGTTGCTAGCATGGCCACGTGGATCGTG-3′

GluClα-AscI reverse 5′-TTGACTGGCGCGCCCTAGCTCAGAACAGAACGTTCTGC-3′

GluClβ-NheI forward 5′-CTAGAGGCTAGCCCACCATGGCCACCCCCT-3′

GluClβ-AscI reverse 5′-TGGATTGGCGCGCCCTACACCAGGGACTCGGGGGT-3′

The GluCl insert was PCR amplified, and the product was digested in NheI and AscI restriction enzymes (NEB) in Cut smart buffer, 37°C 1 hour. The vector plasmid which contained a shortened CAG promoter (shortened cytomegalovirus enhancer fused to the chicken beta-actin promoter, sCAG) with CyRFP in the reverse orientation flanked by NheI and AscI cut sites and heterotypic lox P sites, as well as AAV2 ITR sites (AAV-FLEX-CAG-CyRFP1 was a gift from Ryohei Yasuda, Addgene plasmid # 84357). The CyRFP sequence was removed using NheI and AscI restriction enzymes, 37°C 1 hour. The vector backbone and new inserts were gel extracted and ligated using T4 DNA ligase (NEB), 37°C overnight. The ligation reaction was transformed into competent Ecoli bacteria and plated onto ampicillin agar plates. Ampicillin resistant single colonies were picked and screened for successful plasmid generation. Initial screening was for plasmid size α-7057 bp, β-6973 bp, followed by Sanger sequencing to confirm correct insertion (plasmids made available on Addgene #196995 and #196996). Constructs were commercially packaged and serotyped with the adeno-associated virus (AAV) 9 capsid protein by the Viral Vector Facility (VVF), Neuroscience Center Zurich (ZNZ), University of Zurich and ETH Zurich, to a final titre of 9.8 × 10^12^ vg/mL (vector genomes/mL) AAV9-GluClα.Cre^ON^ and 1.6 × 10^13^ vg/mL AAV9-GluClβ.Cre^ON^.

### 2.3. Cell culture and transfection

#### 2.3.1. Human embryonic kidney 293T cells and transfection

HEK293T cell were routinely cultured in Dulbecco modified Eagle Medium (DMEM, Thermofisher Scientific, United Kingdom) and 10% foetal calf serum. Cells were periodically split using Versene solution (Gibco, Thermofisher Scientific) and mechanical dissociation. Dissociated cells were seeded into 6-well plates and, when cells reached 70% confluence, were transfected using JetPEI following the manufacturers protocol (PolyPlus Transfection, France). A total of 3 μg of DNA/per 35 cm well was combined with NaCl and JetPEI and after 20 minutes added to HEK cells. The next day transfected cells were replated onto cover slips and used 24 hours later.

### 2.4. Dorsal root ganglion neuronal culture

Mice were sacrificed and spinal columns removed. Dorsal root ganglia were rapidly dissected and enzymatically digested at 37°C for 80 minutes in dispase type II (4.7 mg/mL) and collagenase type II (4 mg/mL). Cells were briefly centrifuged and Hank’s balanced salt solution/CollagenaseDispase removed. Prewarmed culture media (Neurobasal, 2% B-27 supplement, 1% Penicillin streptomyocin) was added, and cells were mechanically dissociated using fire-polished pipettes. Cells were plated on poly-D-lysine and laminin-coated cover slips with the addition of growth factors (mouse nerve growth factor [50 ng/mL; NGF, PeproTech, United Kingdom] and 10 ng/mL glial-derived neurotrophic factor [GDNF, PeproTech]).

### 2.5. Dorsal root ganglion electroporation

Neurons were transfected by electroporation using the Neon system (Life Technologies, Thermofisher Scientific). Dissociated cells were resuspended in 10 μL of buffer R plus 1 μg of total plasmid DNA per 50 to 100,000 cells. The electrical protocol applied was 3 1500 V pulses of 10 milliseconds duration. Cells were plated as stated above and used 48 to 72 hours later when plasmid DNA expression was maximal.

### 2.6. Whole-cell patch clamp

Voltage-clamp recordings using an Axopatch 200B amplifier and Digidata 1550 acquisition system (Molecular Devices, UK) were performed at room temperature. Data were sampled at 20 kHz and low-pass filtered at 5 kHz. Series resistance was compensated 70% to 85% to reduce voltage errors. All data were analyzed by Clampfit 10 software (Molecular Devices). GFP/XFP+ DRG neurons and HEK cells were detected with an Olympus microscope with an inbuilt GFP (green fluorescent protein) filter set (470/40x excitation filter, dichroic LP 495 mirror, and 525/50 emission filter). Filamental borosilicate glass capillaries (1.5 mm OD, 0.84 mm ID; World Precision Instruments, United Kingdom) were pulled to form patch pipettes of 2 to 5 MΩ tip resistance and filled with an internal solution containing (mM): 100 K+ gluconate, 28 KCl, 1 MgCl_2_, 5 MgATP, 10 HEPES, and 0.5 EGTA; pH was adjusted to 7.3 with KOH and osmolarity set at 305 mOsm (using glucose). Cells were maintained in a chamber constantly perfused with a physiological extracellular buffer containing (mM): 140 NaCl, 4.7 KCl, 2.5 CaCl2, 1.2 MgCl2, 10 HEPES, and 10 glucose; pH was adjusted to 7.4 with NaOH and osmolarity set at 315 mOsm (using glucose). There was a calculated −13 mV junction potential when using these solutions; voltage values were adjusted to compensate for this. Ivermectin (IVM Sigma Aldrich, United States) was prepared in DMSO (fresh daily), diluted in extracellular buffer to a final concentration of 20 nM and was delivered to the cells by the perfusion system. All post IVM recordings were made 15 minutes after IVM application. Membrane conductance was measured in a voltage-clamp mode with a 100 milliseconds voltage ramp from −90 mV to +40 mV every 10 seconds. The resultant linear current gradient was used to calculate membrane conductance using the rearranged Ohm law equation where V = voltage, I = current, R = resistance, C = conductance. V = IR, = C = 1/R, C = I/V. Resting membrane potential (RMP) was measured in bridge mode (I = 0). In the current clamp mode, DRGs were held at −60 mV. Input resistance was derived by measuring the membrane deflection caused by a 20 pA current step. Rheobase was determined by applying 50 milliseconds depolarising currents of increasing steps of 25 pA until action potential (AP) generation.

For recordings generated following in vivo dosing, IVM (5 mg/kg) was injected in vivo, into mice that had previously received an intrathecal injection of AAV-GluCl.Cre^ON^. Dorsal root ganglions were cultured, and patch-clamp recordings made 24 to 48 hours after IVM dosing.

### 2.7. Surgical intrathecal injection

Heterozygous male and female Nav1.8^Cre^ mice 4 to 8 weeks old were used for intrathecal (i.t.) injection surgery. Each animal was anaesthetised using 2% isoflurane and prepared for surgery by shaving a region over the thoracic vertebrae. After incision site sterilisation (iodine, alcohol wipe), a 1 to 2 cm incision was made to the back (rostral to caudal) above the spine. T-10 and T-11 vertebrae were located; soft tissue was carefully and sparingly removed lateral to the midline to expose the dura and spinal cord. A drop of lidocaine was applied to the dura for approximately 1 to 2 minutes then removed. Using a 30-gauge needle, the dura was carefully punctured (CSF leak at this point suggested a successful puncture). An “in house” developed cannula system was designed by connecting tubing of decreasing size until the final cannula tip measured 0.008 in O.D × 0.004 in I.D. The end of the cannula was inserted approximately 1 cm caudal into the subdural space. Using a syringe pump driver, 8 μL of AAV (AAV9-GluCl.Cre^ON^α and/or AAV9-GluCl.Cre^ON^β— VVF Zurich) was injected into the subdural space at a rate of 1 μL/minute. After injection, the cannula was allowed to rest in position for 2 minutes to avoid back flow and then slowly removed. The dura was coated with a single drop of dura gel (Cambridge NeuroCare) to seal the dura and prevent further CSF leak. Finally, the incision site was sutured closed and appropriate postoperative care and analgesics given (local 2 mg/kg Marcain, AstraZeneca and systemic 5 mg/kg Rimadyl, Pfizer, United Kingdom). Animals were used for behaviour or histology at least 3 weeks postsurgery.

### 2.8. Immunohistochemistry

Animals were deeply anesthetised with pentobarbital, and the blood cleared from all tissues by perfusing saline through the vascular system. Mice were then perfuse fixed using 4% paraformaldehyde (PFA). Lumbar DRGs were collected and postfixed in 4% PFA accordingly for 1 to 2 hours. Dorsal root ganglions were cryoprotected in 30% sucrose for a minimum of 48 hours followed by embedding the tissue and sectioning on a cryostat (12 μm). Cultured cells were fixed with 4% PFA for 10 minutes and treated similarly to other tissues. In brief, samples were washed in PBS and blocked in a blocking solution (5% normal donkey serum, 0.3% TritonX-100, PBS) for 1 hour at room temperature (RT). Primary antibodies (NeuN 1:500, Rabbit, Abcam (Cambridge, United Kingdom) Ab177487. eGFP 1:500, Chicken, Abcam Ab13970. βIII-Tubulin, 1:500, Mouse, R&D Mab1195) were diluted in blocking solution and applied to tissue or cells overnight at RT. The next day samples were washed in a wash solution (0.3% TritonX-100, PBS) followed by a 2-hour incubation with secondary antibodies (Alexa Fluor Pacific Blue, 1:100, Alexa Fluor 488, 1:500, Alexa Fluor 546, 1:500 Thermo Fischer Scientific) diluted in wash solution at RT. Samples were mounted using Vectorshield (with or without DAPI) and imaged on a confocal microscope (Zeiss LSM-710). Images were analysed using Fuji/ImageJ (NIH). For quantification, at least 3 sections per animal were used, with at least 3 animals per group. When detecting GluCl subunits individually, we did not use antibodies, but simply YFP and Cerulean native fluorescence, in all other experiments, we used anti-eGFP (enhanced green fluorescent protein) antibodies to detect both subunits collectively. (eGFP antibodies crossreact and detect YFP and Cerulean).

### 2.9. In situ hybridisation

Dorsal root ganglion and spinal cord in situ hybridisation (ISH) was performed on samples collected from the experimental behavioural cohort. In situ hybridisation was performed by following the user instructions for the RNAScope2.5 RED Chromogenic assay kit or RNAScope Multiplex Fluorescent v2 (Advanced Cell Diagnostics, Bio-techne, United Kingdom). In brief, fixed DRG tissue was pretreated using hydrogen peroxide and a protease treatment. Tissue was next incubated for 2 hours at 40°C with an eGFP mRNA probe (Cat. No. 400281). The GFP probe is also able to detect YFP and Cerulean due to sequence overlap. Next, a series of probe amplification steps were performed followed by a fast red or TSA vivid 520 detection step. Samples were costained and imaged as above. FuJi/Image J was used for analysis. Cells were identified and mRNA intensity calculated; cells were selected as mRNA positive if the intensity was more than the mean + 2 × SD of background intensity. Background intensity was calculated from tissue that underwent that same procedure with a standard negative control mRNA probe (Advanced Cell Diagnostics).

### 2.10. Behavioural testing

Mice (male and female) were tested at a consistent time of day, in the same environment. Mice were habituated to their testing environment and equipment before behavioural test days. The experimenter was blinded to the animal group before testing and until after behavioural analysis was complete. Video analysis (of pinprick and formalin) was carried by a blinded experimenter, to both animal group and the study design. For all behavioural testing, AAV-GluCl.Cre^ON^αβ injected mice were used as the experimental group and AAV-GluCl.Cre^ON^β-only injected mice were used as controls. Behaviour testing was performed on adult mice 4 to 6 weeks after AAV injection. For all mechanical testing, mice were randomly chosen from their home cage and assigned a test box (5 × 5 × 10 cm) which was elevated on a wire mesh base and were acclimatised to the equipment for 30 to 60 minutes. All behavioural tests were conducted to achieve a baseline then repeated once 24 hours after IVM (Noromectin 1.0% wt/vol) administration (0.5 µL/g of ready to use Noromectin equates to 5 mg/kg as in Weir et al. 2017), unless otherwise stated.

von Frey: Mice were then tested on their plantar hind paws using calibrated von Frey hairs (Linton Instrumentation) using the “updown” method to evaluate their 50% paw withdrawal thresholds. Mice were tested on 3 different days to obtain an average baseline value.

Brush or cotton swab: Similar to,^14,29^ the plantar hind paws of mice were brushed (1 cm/second) with a fine artists paint brush or a cotton swab that had been puffed out to 3 times its original size. Each mouse received 5 successive stimuli on alternate hind paws (10 seconds apart) twice. The number of responses was recorded. A response included lifting, flicking, or moving the hind paw or walking away from the stimulus. Mice were tested on 3 different days to obtain an average baseline value.

Pinprick: Mice were tested on their plantar hind paws using a sharp pin attached to a 1-g calibrated von Frey filament.^3^ Mice were video recorded using a smart phone at 120 fps (8.33 ms per frame), and the latency to withdraw from the pinprick analysed, 3 times per paw, on 3 different days.

Tape test: Similar to,^50^ a small 1 cm by 1 cm piece of tape was placed onto the hind limb of mice, and the time taken from mice to detect the tape was measured (sense time), on 3 different days to obtain a baseline.

53°C hot plate: Mice were chosen at random from their home cage and placed onto a Perspex-enclosed hot plate (UgoBasile) and were observed until mice displayed pain behaviours on their hind paws, ie, lifting, flicking, and licking of the hind paw (cut off 30 seconds to prevent tissue damage). The latency to respond was recorded, and mice were tested on 3 different days to obtain average baseline value.

Rotarod: Mice were chosen at random from their home cage and briefly acclimatised to the rotarod apparatus (Ugo Basile, 47600). Mice were placed onto the rotarod equipment while the central rod rotated at a speed of 32 rpm. The latency to fall was recorded on 2 different days to achieve a baseline.

### 2.11. Chemical pain assay—formalin injection

Mice were chosen at random from their home cage, and the left hind paw of each mouse was injected subcutaneously with 2% formalin, and mice were immediately placed in a test box (5 × 5 × 10 cm), on a glass base, which was elevated above a camera. The perimeter and roof of the test box consisted of mirrors to allow good visualisation of the injected hind paw. The mice were video recorded in this environment for 1 hour while the experimenter left the room. Off line analysis was used to measure nocifensive behaviours of the injected hind paw (lifting, licking, flinching, and shaking) every 5 minutes for 60 minutes. The formalin assay was also analysed in 2 phases: first phase 0 to 15 minutes and second phase 15 to 60 minutes. All formalin behaviour was conducted 24 hours after IVM injection.

### 2.12. Sample sizes and statistical analysis

Behavioural sample sizes were calculated using the software G*power2 with *P*-values of 0.05 and a power of >0.8. In pain behaviour outcomes, the effect size is taken as a 30% difference which parallels what is often seen as clinically relevant changes in pain ratings in human. Data on variance were generated from our provisional and published data using these outcome measures. The primary outcome was evoked behaviour, experimental unit was mouse, units per group was 9 biological replicates, 3 measurements, effect size 30 (f = 0.6), and RM analysis of variance (ANOVA) was chosen for the primary outcome statistical test. Note that for group sizes in cohorts that underwent AAV intrathecal injection n of 1 was added to this sample size to deal with surgical attrition. All data were tested for normality using the D'Agostino–Pearson normality test and the appropriate parametric or non-parametric statistical tests used accordingly. All statistical tests used were 2 tailed. Statistical comparisons were made using a Student *t* test or Mann–Whitney *U* test. In experimental groups in which multiple comparisons and repeated measures (RM) were made, 2-way ANOVA tests with appropriate post hoc tests were performed. All data are represented as mean ± the standard error of the mean (SEM) unless otherwise stated. Statistical significance is indicated as follows *P < 0.05, **P < 0.01, ***P < 0.001, ****P < 0.0001. The statistical test used is reported in the appropriate figure legend. GraphPad prism 9 was used to perform statistical tests and graph data. Adobe illustrator CS5 was used to create schematics, and medical graphics were obtained from Smart servier free medical art (smart.servier.com).

## 3. Results

### 3.1. Generation and validation of GluClαβ.Cre^ON^ in vitro

**Figure 1.**
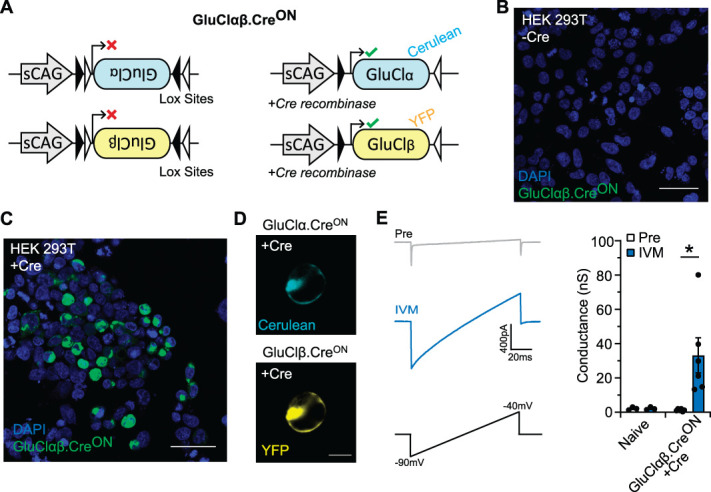
Functional GluClαβ.Cre^ON^ in HEK293T cells after exogenous Cre-recombinase expression. (A) Schematic of GluCl.Cre^ON^ design. Left: inverted GluClα or GluClβ flanked by heterotypic lox site with a short CAG promoter. Right: Example of cre-recombinase mediates inversion of GluCl and subsequent GluCl subunit expression. (B and C) HEK293T cells transfected with GluClαβ.Cre^ON^ in the absence of cre-recombinase (B), or with cre-recombinase (C). GluClαβ.Cre^ON^ expression was only detected in HEK293T cells when cotransfected with cre-recombinase. (D) HEKT293T cell transfected with GluClαβ.Cre^ON^ and imaged to visualise each tagged subunit (GluClα-Cerulean and GluClβ-YFP). Each subunit can be recognised as membrane localised. (E) Transfected HEK293T cells expressing GluClαβ.Cre^ON^, but not naïve cells, show a significant chloride conductance after 20 nM IVM. Left: Example currents before and after IVM. Right: Quantification of membrane conductance before and after IVM (Naïve n = 3 cells, GluClαβ.Cre^ON^ +Cre n = 6 cells, RM-2 way ANOVA with the Bonferroni post hoc test before vs after IVM, t = 3.74, df = 7, *P* = 0.014, *). All data mean ±SEM. Scale bar 50 µm. ANOVA, analysis of variance; GluCl, glutamate-gated chloride channel; IVM, ivermectin.

**Figure 2.**
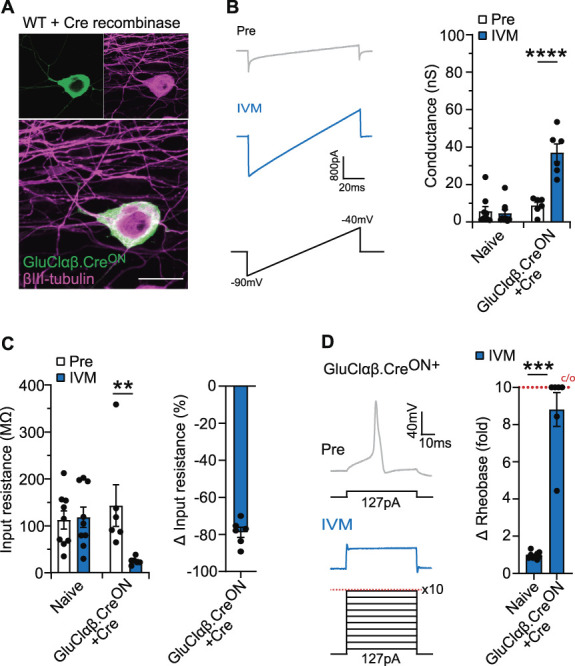
Functional GluClαβ.Cre^ON^ in sensory neurons after Cre-recombinase cotransfection. (A) Example image of a GluClαβ.Cre^ON^ transfected wild-type DRG neuron that was cotransfected with Cre-recombinase. (B) 20 nM IVM induced a significant chloride conductance in GluClαβ.Cre^ON^ + Cre-transfected sensory neurons but not naïve neurons. Left: example currents before and after IVM. Right: Quantification of membrane conductance before and after IVM (naïve n = 9 neurons, GluClαβ.Cre^ON^ + Cre n = 6 neurons, RM-2 way ANOVA with the Bonferroni post hoc test before vs after IVM, t = 11.55, df = 13, *P* < 0.0001, ****). (C) IVM caused a significant reduction in membrane resistance of GluClαβ.Cre^ON^ + Cre but not naïve neurons (naïve n = 9 neurons, GluClαβ.Cre^ON^ + Cre n = 6 neurons, RM-2 way ANOVA with Bonferroni post-hoc test pre vs post IVM, t = 4, df = 13, *P* < 0.003, **). The change in membrane resistance of GluClαβ.Cre^ON^ neurons was also quantified as a percentage change (right). (D) IVM reduced the excitability of GluCl.Cre^ON^ expressing neurons. The Rheobase was recorded before and after IVM. Left: example trace of an action potential, its current threshold pre IVM, and the loss of action potential firing after IVM. Right: change in Rheobase was calculated for naïve and GluClαβ.Cre^ON^ + Cre neurons, with a 10 times increase in threshold set as the cut off. 1 = no change. (Naïve n = 9 neurons, GluClαβ.Cre^ON^ + Cre n = 6 neurons, Mann–Whitney test, *P* = 0.0004, ***). All data mean ±SEM. Scale bar 25 µm. ANOVA, analysis of variance; DRG, dorsal root ganglion; GluCl, Glutamate-gated chloride channel; IVM, ivermectin.

There is an expanding repertoire of cre-recombinase driver lines that, in combination with cre-dependent actuator constructs, can be used to genetically target molecularly defined populations of sensory neurons. We designed and generated novel GluCl constructs where the open reading frame of α (Cerulean tagged) and β subunits (YFP tagged) (functional GluCl requires both subunits) were inverted and flanked by heterotypic LoxP sites (Fig. 1A). Cre-mediated inversion recombination is required for correct transcription, facilitating selective expression (Fig. 1A). We expressed our newly generated GluClαβ.Cre^ON^ tool in HEK293T cells and only observed expression when Cre was also present (Figs. 1B,C). In addition, as GluCl.Cre^ON^ subunits are independently tagged, we were able to confirm that both subunits trafficked to the cell membrane after cre-mediated recombination (Fig. 1D). We next performed voltage-clamp recordings of GluClαβ.Cre^ON^ and Cre-recombinase expressing HEK293T cells and confirmed that GluClαβ.Cre^ON^ was functional, as shown by the potent agonist IVM inducing a significant chloride conductance (33.17 ± 10.22 nS) compared with pretreatment (1.29 ± 0.26 nS) (Fig. 1E). We also recorded the conductance from naïve HEK293T cells before and after IVM to confirm that GluClαβ.Cre^ON^ cells do not exhibit a Cl-conductance before IVM. To test our GluClαβ.Cre^ON^ system in sensory neurons, we cultured mouse dorsal root ganglion neurons and coelectroporated them with GluClαβ.Cre^ON^ and a Cre-recombinase expressing plasmid and observed GluClαβ.Cre^ON^ expression (Fig. 2A). Importantly, GluClαβ.Cre^ON^ was not detected in wild-type cultures without cre-recombinase (Supplemental Fig. 1A-B, available at http://links.lww.com/PAIN/B868). GluClαβ.Cre^ON^ positive cells were visually selected, and voltage-clamp recordings confirmed that low-dose (20 nM) IVM induced a significant chloride conductance (Fig. 2B). Ivermectin did not induce a conductance in naïve sensory neurons (Fig. 2B). The same neurons were recorded in a current-clamp mode to assess sensory neuron excitability pre-IVM and post-IVM. Application of low-dose IVM on GluClαβ.Cre^ON^ neurons reduced input resistance by 78.83 ± 2.74% (Fig. 2C) and increased the minimum current required to elicit and action potential (rheobase) post-IVM by 4.4 to >10-fold (Fig. 2D). Ivermectin treatment to naïve sensory neurons did not alter input resistance or excitability.

**Figure 3.**
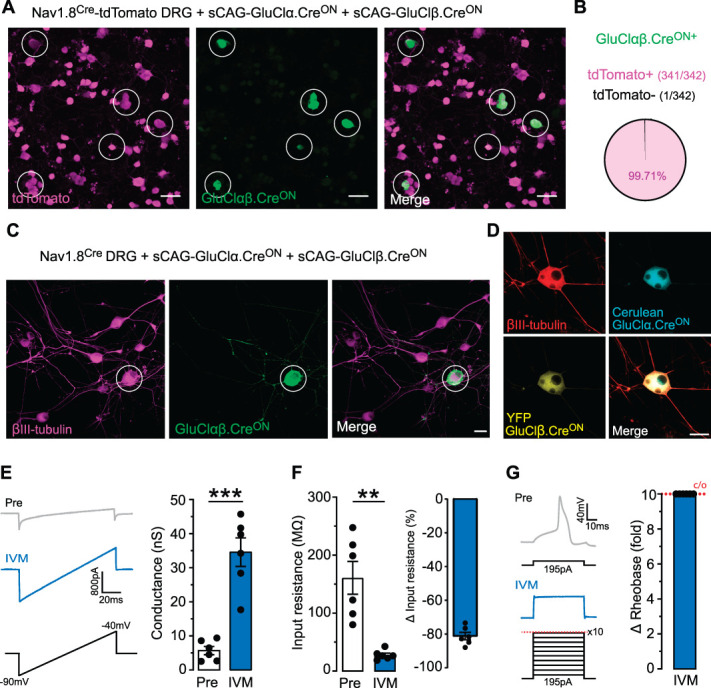
GluClαβ.Cre^ON^ can silence nociceptors that constitutively express Cre-recombinase. (A) GluClαβ.Cre^ON^ was transfected into DRG neurons from Nav1.8^Cre^TdTomato (Nav1.8^Cre+/-^Ai14^+/-^) mice. GluClαβ.Cre^ON^ expression highlighted by white circles. Scale bar 50 µm. (B) 341/342 GluClαβ.Cre^ON^ positive neurons coexpressed tdTomato. (C) Nav1.8Cre sensory neurons transfected with GluClαβ.Cre^ON^. (D) Each GluClαβ.Cre^ON^ subunit can be visualized in sensory neurons using tag fluorescence (E) GluClαβ.Cre^ON^ positive neurons exhibited a significant chloride conductance after 20 nM IVM. Left: examples currents before and after IVM. Right: quantification of membrane conductance before and after IVM (n = 6 neurons, paired *t* test, t = 7.194, df = 5, *P* = 0.0008, ***). (F) The membrane resistance of GluClαβ.Cre^ON^ expressing neurons was significantly reduced after IVM (n = 6 neurons, paired *t* test, t = 5.188, df = 5, *P* = 0.0035, **). This change in membrane resistance was also quantified as a percentage (right). (G) IVM rendered GluClαβ.Cre^ON^ expressing neurons silent. The Rheobase was recorded before and after IVM. Left: example trace of an action potential, its current threshold pre IVM, and the loss of action potential firing post IVM. Right: change in Rheobase was calculated with a 10 times increase in Rheobase set as the cut off. All data mean ± SEM. Scale bar 25 µm. DRG, dorsal root ganglion; GluCl, glutamate-gated chloride channel; IVM, ivermectin.

Next, our tool required validation in a neuronal system where Cre-recombinase is constitutively expressed. We took advantage of the Nav1.8^Cre^ line that has been well characterised and is known to express Cre-recombinase in nociceptors and C-low threshold mechanoreceptors.^2,15,29,32^ We labelled Cre-expressing sensory neurons by crossing Nav1.8^Cre^ mice with the Cre-dependent tdTomato reporter line Ai14. Sensory neurons from Nav1.8^Cre^;Ai14 mice were isolated and transfected with GluClαβ.Cre^ON^. As expected GluCl expression was highly selective for tdTomato positive neurons (99.71%) (Figs. 3A,B). We expressed GluClαβ.Cre^ON^ in sensory neurons from Nav1.8^Cre^-positive mice (Fig. 3C) and were able to detect each subunit independently (Fig. 3D). We performed voltage-clamp recordings to test the function of GluClαβ.Cre^ON^ in Nav1.8^Cre^-positive sensory neurons and observed a large IVM-induced chloride conductance (Fig. 3E). As expected, this conductance was accompanied by a large decrease in the membrane input resistance (Fig. 3F), and all recorded neurons were rendered fully silent (Fig. 3G). Collectively, these results demonstrate the functional validity of a Cre^ON^ system for the selective expression of the GluCl chemogenetic silencer in vitro.

### 3.2. The expression and function of AAV-GluCl.Cre^ON^ in nociceptors

**Figure 4.**
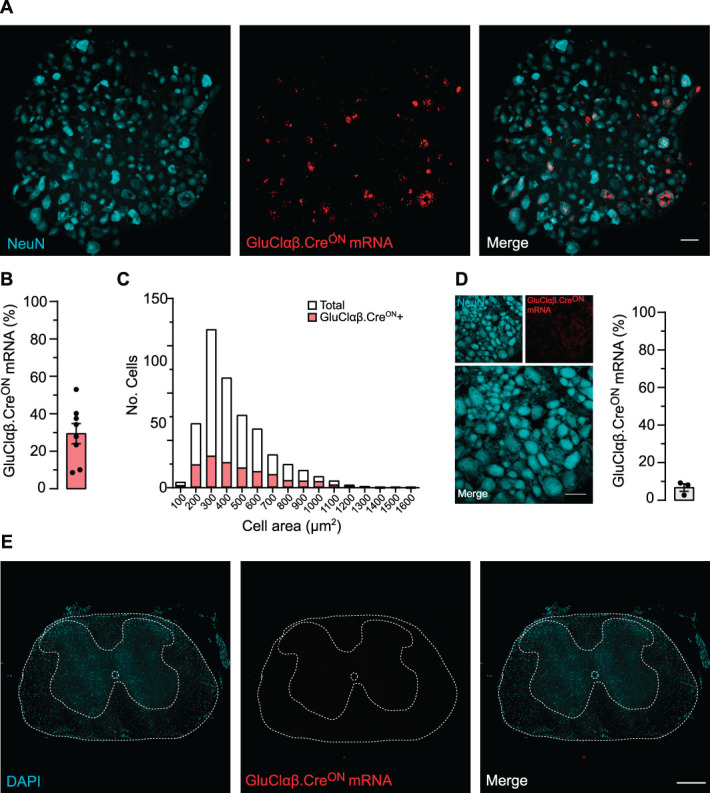
AAV-GluClαβ.Cre^ON^ expression selectively in the DRG after intrathecal delivery. (A) In situ hybridisation was used to identify GluClαβ.Cre^ON^ mRNA expression in the DRG after AAV injection. (B) GluClαβ.Cre^ON^ mRNA was detected in 29.4 ± 5.35% of DRG neurons from Nav1.8^Cre^ mice (n = 8 mice, 2414 cells) (C) Cell size distribution of GluCl.Cre^ON^ mRNA-positive neurons. (D) GluClαβ.Cre^ON^ shows a low level of leak expression, <10%, when AAV- GluClαβ.Cre^ON^ was injected into wild-type mice. Very low levels of GluCl.Cre^ON^ mRNA was detectable in the DRG (n = 3 mice, 1181 cells). (E) Spinal cord section from Nav1.8Cre AAV-GluClαβ.Cre^ON^-injected mice. GluClαβ.Cre^ON^ mRNA was absent from the spinal cord. All scale bars (A–D), 50 μm (E) 300 µm. All data mean ± SEM. DRG, dorsal root ganglion; GluCl, glutamate-gated chloride channel.

The aim for this tool is to facilitate the selective targeting and silencing of sensory neuron circuits using Cre driver lines in vivo. To target nociceptors, we generated adeno-associated virus (AAV) particles containing GluClα.Cre^ON^ and GluClβ.Cre^ON^. To test our viruses in vitro, we treated Nav1.8^Cre^ or wild type neurons with both AAVs. We saw GluClαβ.Cre^ON^ expression only in sensory neurons from Nav1.8^Cre^ mice and not wild-type neurons (Supplemental Fig. 2A-D, available at http://links.lww.com/PAIN/B868). This was encouraging that our system had specificity, so we next moved in vivo. We intrathecally injected AAV9.sCAG.GluClαβ.Cre^ON^ (one virus for each subunit, see methods) into Nav1.8^Cre^ mice and examined GluClαβ.Cre^ON^ expression at least 4 to 6 weeks later. When using immunohistology to detect the YFP or Cerulean-tagged GluCl subunits, we observed low levels of expression per cell which made classification of expressing/nonexpressing difficult, so we switched to an in situ hybridisation approach. We used RNAscope to detect the tag-mRNA within the GluClαβ.Cre^ON^ transcripts and saw an expression rate of 29.4 ± 5.35% of total DRG neurons (Figs. 4A,B). We performed cell size analysis and confirmed that GluClαβ.Cre^ON^ mRNA was detected in small–medium-sized neurons as expected (Fig. 4C). To test for leak expression, AAV-GluClαβ.Cre^ON^ was injected into wild-type mice and DRGs sampled for GluClαβ.Cre^ON^ mRNA expression. We saw some leak expression albeit at a low level (Fig. 4D). To investigate AAV-GluClαβ.Cre^ON^ selectivity in more detail, we i.t. injected Nav1.8^Cre^tdTomato reporter mice and analysed GluClαβ.Cre^ON^ and reporter coexpression. We saw that >80% of GluClαβ.Cre^ON^ positive neurons coexpressed tdTomato. (Supplemental Fig. 3A-B, available at http://links.lww.com/PAIN/B868). Finally, using our intrathecal injection method, we did not target spinal neurons; GluClαβ.Cre^ON^ mRNA was absent in the spinal cord (Fig. 4E). Together, we show that AAV-GluClαβ.Cre^ON^ can be used to selectively target neuronal populations in vivo, in a selective Cre-dependent manner.

**Figure 5.**
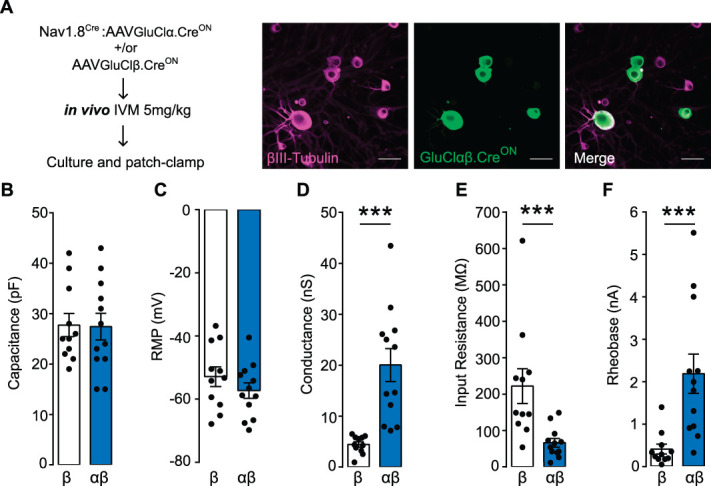
Functional silencing of AAV-GluCl.Cre^ON^ expressing neurons, after in vivo IVM dosing. (A) AAV- GluClαβ.Cre^ON^ subunits were intrathecally injected into Nav1.8^Cre^ mice. Four weeks after AAV injection mice were dosed in vivo with 5 mg/kg IVM. DRG neurons were cultured 24 hours later and patch-clamp performed within 36 hours. Example image of GluClαβ.Cre^ON^ + cultured DRG neurons. Scale bar 25 µm. (B) The recorded capacitance of either Nav1.8^Cre^GluCl.Cre^ON^.βonly (white) or Nav1.8^Cre^GluCl.Cre^ON^αβ (blue) neurons. The cell capacitance was on average the same for each group (unpaired *t* test, n = β 11 cells, αβ 12 cells, *t* = 0.088 df = 21, *P* = 0.930). (C) The resting membrane potential (RMP) was not significantly different in either Nav1.8^Cre^GluCl.Cre^ON^.βonly or Nav1.8^Cre^GluCl.Cre^ON^αβ neurons (unpaired *t* test, n = β—11 cells, αβ—12 cells, *t* = 1.125, df = 21, *P* = 0.272). (D) Nav1.8^Cre^GluCl.Cre^ON^αβ neurons had a significantly higher conductance than β-only expressing neurons after in vivo IVM dosing (unpaired *t* test, n = β 11 cells, αβ 12 cells, *t* = 4.562, df = 21, *P* = 0.0002, ***). (E) The input resistance of Nav1.8^Cre^GluCl.Cre^ON^αβ expressing neurons was significantly lower than β-only expressing neurons after IVM dosing in vivo (Mann–Whitney *U* test, n = β 11 cells, αβ 12 cells, *U* = 13, *P* = 0.0005, ***). (F) After IVM dosing in vivo, the rheobase of Nav1.8^Cre^GluCl.Cre^ON^αβ-expressing neurons was significantly higher than β-only expressing neurons (Mann–Whitney *U* test, n = β 11 cells, αβ 12 cells, *U* = 10, *P* = 0.0002, ***). All data mean ±SEM. DRG, dorsal root ganglion; GluCl, glutamate-gated chloride channel; IVM, ivermectin.

Silencing efficacy of optogenetic or chemogenetic actuators are often tested indirectly by applying light sources or agonists in vitro. The slow off kinetics of IVM-induced GluCl activation (at least 3 days^49^) allowed us to validate silencing under in vivo dosing conditions. Sensory neurons were cultured from AAV-GluClαβ.Cre^ON^ injected mice which had received an intraperitoneal injection of 5 mg/kg IVM 12 to 16 hours prior. Patch-clamp recordings from dissociated GluClαβ.Cre^ON^-positive neurons were subsequently performed 12 to 24 hours later to assess silencing efficacy (Fig. 5A). For these and all following experiments, our experimental group received injections of both GluCl.Cre^ON^ subunits α and β, and our control group consisted of injecting only the β-only subunit of GluCl.Cre^ON^. We have previously shown that the expression of GluCl β subunit alone is not sufficient to induce a functional IVM induced chloride conductance and cannot suppress neuronal excitability.^49^ We measured the cell capacitance of recorded GluClαβ.Cre^ON^+ neurons, and it was comparable between groups and unaffected by an IVM-induced chloride conductance (Fig. 5B). Cell capacitance is well correlated to cell size, and our GluClαβ.Cre^ON^ neurons had capacitance values one would expect for small to medium-sized sensory neurons, reflecting the size profile of the Nav1.8+ sensory neurons. We observed that following in vivo IVM treatment, the resting membrane potential was not significantly different between GluClαβ.Cre^ON^ or β-only expressing neurons (Fig. 5C). We confirmed using voltage-clamp recordings that GluCl.Cre^ON^ αβ neurons exhibited a significant background conductance (that resembles a chloride conductance), compared with GluCl.Cre^ON^ β-only control neurons (Fig. 5D). Similar to above, in vivo IVM dosing led to GluCl.Cre^ON^ αβ neurons exhibiting a significantly lower membrane input resistance, and a significantly higher rheobase compared with our β-only control neurons (Figs. 5E,F). These data demonstrate that AAV-GluCl.Cre^ON^ can be used to functionally silence sensory neuron subpopulations in vivo.

### 3.3. Selective silencing of pain-related behaviours

**Figure 6.**
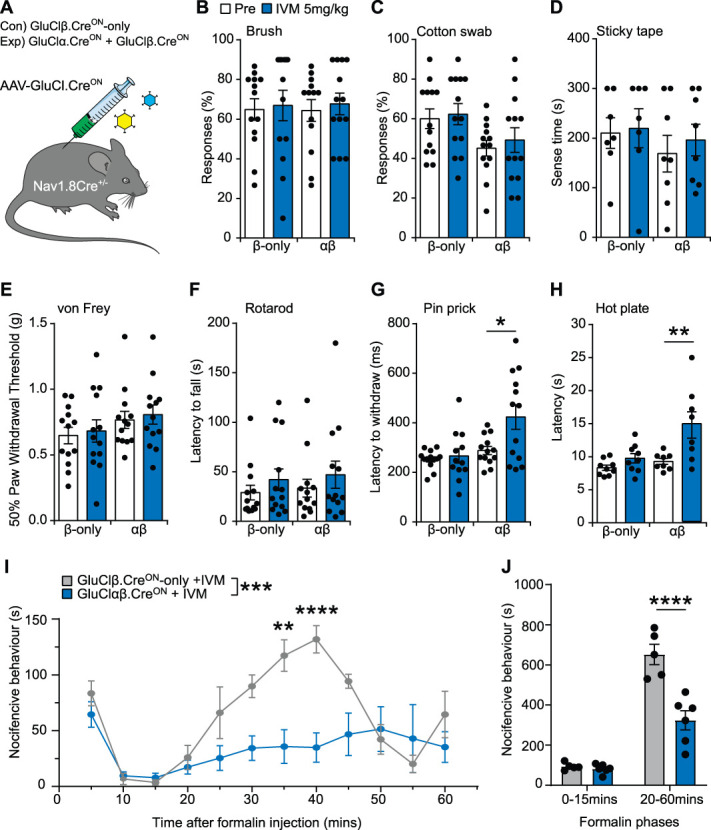
Using AAV-GluClαβ.Cre^ON^ in Nav1.8^Cre^ mice can selectively silence mechanical, thermal, and chemical pain. (A) Schematic of the experimental design. Nav1.8^Cre^ mice were injected with AAV-GluCl.Cre^ON^ α or β and underwent behavioural testing before and after IVM. (B) There were no significant changes in the mechanical detection of light touch brush in either Nav1.8^Cre^GluCl.Cre^ON^ β-only control mice or Nav1.8^Cre^GluCl.Cre^ON^ αβ mice after IVM (RM 2-way ANOVA, with Bonferroni post hoc test before vs after IVM, β-only; n = 13 mice, t = 0.347, df = 24, *P* > 0.99, αβ; n = 13 mice, t = 0.565, df = 24, *P* > 0.99). (C) There were no significant changes in the mechanical detection of light touch cotton swab in either Nav1.8^Cre^GluCl.Cre^ON^ β-only control mice or Nav1.8^Cre^GluCl.Cre^ON^ αβ mice after IVM (RM 2-way ANOVA, with Bonferroni post hoc test before vs after IVM, β-only; n = 13 mice, t = 0.562, df = 24, *P* > 0.99, αβ; n = 13 mice, t = 0.99, df = 24, *P* = 0.655). (D) There were no significant changes in the mechanical detection of sticky tape in either Nav1.8^Cre^GluCl.Cre^ON^ β-only control mice or Nav1.8^Cre^GluCl.Cre^ON^ αβ mice after IVM (RM 2-way ANOVA, with the Bonferroni post hoc test before vs after IVM, β-only; n = 7 mice, t = 0.191, df = 13, *P* > 0.99, αβ; n = 8 mice, t = 0.58, df = 13, *P* > 0.99). (E) There were no significant changes in the mechanical detection of von Frey in either Nav1.8^Cre^GluCl.Cre^ON^ β-only control mice or Nav1.8^Cre^GluCl.Cre^ON^ αβ mice after IVM (RM 2-way ANOVA, with Bonferroni post hoc test before vs after IVM, β-only; n = 13 mice, t = 0.356, df = 24, *P* > 0.99, αβ; n = 13 mice, t = 0.42, df = 24, *P* > 0.99). (F) Silencing Nav1.8 positive primary afferents did not affect motor function (RM 2-way ANOVA, with Bonferroni post hoc test before vs after IVM, β-only; n = 13 mice, t = 1.80, df = 24, *P* = 0.165, αβ; n = 13 mice, t = 1.87, df = 24, *P* = 0.146). (G) Nav1.8^Cre^GluCl.Cre^ON^ αβ mice exhibited significant hyposensitivity to a noxious pinprick after IVM. (RM 2-way ANOVA, with Bonferroni post hoc test before vs after IVM, β-only; n = 13 mice, t = 1.157, df = 24, *P* = 0.517, αβ; n = 13 mice, t = 2.87, df = 24, *P* = 0.016,*). (H) Nav1.8^Cre^GluCl.Cre^ON^ αβ mice exhibited significant hyposensitivity to a noxious 53°C hotplate after IVM. (RM 2-way ANOVA, with Bonferroni post hoc test before vs after IVM, β-only; n = 9 mice, t = 1.150, df = 15, *P* = 0.464, αβ; n = 8 mice, t = 4.15, df = 15, *P* = 0.0017, **). (I) Silencing Nav1.8 positive primary afferents using GluClαβ.Cre^ON^ and IVM significantly reduced formalin-evoked nocifensive behaviour compared with control mice (RM 2-way ANOVA, β-only; n = 5 mice, vs αβ; n = 6 mice, F (1, 9) = 23.81, *P* = 0.0009, ***, with Bonferroni post hoc test, 35 minutes t = 4.019, df = 108, *P* = 0.0013, **, 40 minutes, t = 4.782, df = 108, *P* < 0.0001, ****). (J) The formalin response can be divided into 2 phases. Nav1.8^Cre^ GluClαβ.Cre^ON^ + IVM mice exhibited significantly less nocifensive behaviour during the second phase of formalin compared with β-only control mice. (RM 2-way ANOVA, with Bonferroni post hoc test β-only n = 5 mice vs αβ n = 6 mice, first phase; t = 0.236, df = 18, *P* > 0.99, second phase; t = 6.54, df = 18, *P* < 0.0001, ****). All data mean ± SEM. ANOVA, analysis of variance; GluCl, glutamate-gated chloride channel; IVM, ivermectin.

Following our generation and validation both in vitro and in vivo, we aimed to test if selectively expressing GluCl in Nav1.8 positive would allow us to chemogenetically silence nociceptors and pain-related behaviours. We injected Nav1.8^Cre^ mice with AAV-GluClαβ.Cre^ON^ or β only (Fig. 6A) and conducted a battery of acute sensorimotor tests before and 24 hours after IVM dosing. We saw no changes in light touch-associated behaviours, which included brush, cotton swab, sticky tape, and von Frey (Figs. 6B,E). We also confirmed that motor function was preserved in each group after IVM dosing; we saw no changes in the latency to fall from a Rotarod (Fig. 6F). However, when assessing the latency to withdraw from a noxious pinprick or the latency to respond to a 53°C hot plate, GluClαβ.Cre^ON^ expressing Nav1.8^Cre^ mice responded significantly slower compared with pre-IVM (Figs. 6G,H). Finally, we wanted to test our system in a model of chemical pain that elicits a persisting affective pain response. We chose the formalin pain model that has 2 classical phases and after administration results in lifting, biting, flinching, licking, and attending to the afflicted paw region. We injected formalin into the hind paws of AAV-GluClαβ.Cre^ON^ or β-only expressing Nav1.8^Cre^ mice that had been predosed IVM 24 hours prior and quantified the nocifensive behaviours over time. We observed that compared with control mice, AAV-GluClαβ.Cre^ON^-expressing Nav1.8^Cre^ mice exhibited significantly less nocifensive behaviour, which was particular apparent during the second phase of the formalin model (Figs. 6I,J).

Taken together, these data show that when selectively targeting nociceptors using our GluClαβ.Cre^ON^ system in Nav1.8^Cre^ mice, we could selectively silence mechanical, thermal, and chemical pain, while preserving nonnociceptive behaviours such as light touch and motor function.

## 4. Discussion

We aimed with this study to expand the chemogenetic toolbox and generate new versions of the engineered GluClv2.0 to selectively interrogate pain circuits. We developed and validated GluClαβ.Cre^ON^ as a selective chemogenetic silencer using the cre/lox system. As a proof of concept, Nav1.8^Cre^ mice received AAV-GluClαβ.Cre^ON^ to target and suppress nociceptor activity and reduce pain-related -vectors are capable of directing selective transgene expression when a cell-type promoter is incorporated or by taking advantage of cre/Lox technology.^45^ The former method comes with significant challenges, such that promoters of applicable size that drive powerful and specific expression are rare, Cre/Lox technology overcomes these 2 issues; one can drive transgene expression in desired populations using a short ubiquitous promoter (such as shortened CAG). The Cre/LoxP system greatly depends on the availability of Cre-expressing mouse driver lines, which in the sensory biology field is becoming more readily available, aided by ever improved knowledge on the molecular make-up of discrete populations.^37^

The aim was to take advantage of the Cre or Lox system and generate novel GluCl constructs which depend on Cre recombinase for expression.^5,38^ GluClαβ.Cre^ON^ was created, where GluCl subunits were cloned in the reverse orientation and flanked by heterotypic Lox P sites. In the presence of Cre recombinase, this results in inversion recombination of the GluCl transgene to the correct reading frame and selective GluCl expression. This method was chosen over the alternative method of integrating a Lox STOP Lox coding region before the GluCl transgene. The latter approach was not possible due to viral packaging limits. The generated GluClαβ.Cre^ON^ construct was validated in vitro both using immunohistology as well as a patch-clamp analysis. GluClαβ.Cre^ON^ showed selective expression only in cells coexpressing Cre-recombinase and was functionally validated in 3 systems: (1) HEK293T cells cotransfected with Cre, (2) wild-type DRG neurons cotransfected with Cre, and (3) DRG neurons cultured from Nav1.8^Cre^ mice which constitutively express Cre-recombinase in the Nav1.8 positive population. In all cases, GluCl, when activated by low doses of IVM, was able to induce a large chloride conductance and silence neuronal activity.

The Nav1.8^Cre^ line used herein is often believed of as a nociceptor-specific mouse line, but it has been further characterised and shown to label both nociceptors and C-LTMRs,^29,40^ which should be considered when interpreting our results. We showed that GluClαβ.Cre^ON^ was functional when virally expressed in Nav1.8-positive neurons and IVM given in vivo. Neurons were subsequently cultured and assessed using patch clamp. Although silencing does not alter RMP, αβ-expressing neurons exhibited a large chloride conductance, low input resistance and increased rheobase. On average, the rheobase was 5.4x larger compared with β-only control expressing neurons. Although this increase was large, it was not as large as the excitability changes seen with in vitro treatment. This could be due to differences in IVM given in vitro vs in vivo*,* partial IVM clearance, or a lowered level of GluCl expression after viral transduction in comparison with DNA electroporation. Unlike the original GluClv2.0,^49^ GluClαβ.Cre^ON^ requires successful viral transduction and Cre-mediated recombination of each subunit, potentially reducing expression efficiency. GluClαβ.Cre^ON^ was also designed using a shorter CAG promoter which could account for the variation between GluClv2.0 and GluClαβ.Cre^ON^ expression. Indeed, levels of GluCl protein detected by immunohistochemistry were low in comparison with previous studies using AAV to transduce DRG neurons. However, when quantifying mRNA levels, we saw approximately 30% of sensory neurons expressed GluClαβ.Cre^ON^ (the Nav1.8 population accounts for 60-70% of the DRG). It should also be considered when interpreting our results that we saw a small amount of leak expression when using our system in vivo*.* This was likely due to spontaneous recombination of our viral vectors, which is a common problem with Cre-dependent viral systems.^17^ We also concede that efficient transgene expression may depend on the strength of the Cre line and the number of viral copies that transduce each neuron, factors that are challenging to control. Future work will investigate how to increase this Cre-dependent expression of GluClαβ.Cre^ON^ using alternative promoters and novel viral vectors.^8,9^

The GluCl.Cre^ON^ system offered high silencing capability, despite the relatively low levels of transgene expression. The 5.4x increase in rheobase using our GluClαβ.Cre^ON^ system in vivo can be interpreted as physiologically relevant and certainly in excess of that demonstrated for both DREADD and optogenetic silencing of sensory neurons.^35,36^ We assessed acute behavioural responses before and after Nav1.8+ population silencing and confirmed that our observed silencing was enough to produce hyposensitivity to noxious heat and noxious mechanical stimuli, but not light touch sensibility or motor function.

Our results are in contrast to a study which ablated (using a diphtheria toxin system) the Nav1.8 positive population.^1^ Abrahamsen et al. (2008) did not observe changes in thermal nociception. Our work also contrasts the optogenetic silencing of the Nav1.8 population; the study did not observe changes in heat pain in naïve mice, although likely due to the technical limitations of optoillumination.^11^ It is surprising that these 2 studies did not see changes in heat nociception, when nociceptors are known to express the TRP channel triad responsible for noxious heat sensing.^47^ In accordance with our results, ablation or inactivation of the TRPV1-lineage or CGRPα populations (which are both Nav1.8 positive) resulted in heat hyposensitivity.^10,31^ Collectively, this may highlight issues with using different strategies to investigate the function of sensory neuron populations. For instance, ablation studies have provided valuable insight into sensory neuron heterogeneity and population function. However, cautious consideration in needed when interpreting these data for the following reasons. Ablation using the diphtheria toxin system is irreversible and in itself could be considered an injury. It is also unclear how the anatomical loss of a population might after the function of other populations and their networks within the CNS. In particular, ablation could lead to synaptic plasticity in the spinal cord dorsal horn or an immune response or microglia recruitment related to the cell death.^18^ This is particularly important as there is strong interplay between the immune system and pathological pain.^6^ Equally, the optogenetic silencing by Daou et al. (2016) used a tool which extruded protons for 1 hour. Increasing the extracellular proton concentration for 1 hour would likely have other consequences and may activate acid-sensing ion channels (ASICs) and other pH sensitive proteins. Indeed, heterologously expressed ASICs were activated by yellow light or Arch-mediated proton efflux.^52^ Furthermore, computational models predicted the peak proton concentration at the extracellular membrane, as a result of Arch activation would reach pH 6.7, enough to activate ASICs in vivo*.*^52^ This is particularly pertinent as ASICs are known to modulate mechanosensory function.^33^ We believe our system circumvents issues surrounding ablation and pH. Other chemogenetic systems that may circumvent these issues include the PSAM/PSEM modular systems.^25,26^ In particular, the most recent iteration, PSAM^4^-GlyR, can be used to silence neuronal activity using ultrapotent designer agonists.^26^ As a single vector strategy, this may offer benefits over GluCl. However, the potency of PSAM^4^-GlyR silencing has yet to be tested in sensory neurons.

Finally, we used our system to silence the Nav1.8 positive population while measuring the nocifensive response to an injection of formalin. This is a chemical pain assay believed to resemble inflammatory-like tonic pain.^44^ When using GluClαβ.Cre^ON^ to silence nociceptive afferents, the response in the second phase of the formalin assay was significantly reduced. This aligns with Abrahamsen et al.,^1^ who also saw abolishment of formalin evoked inflammatory-like pain in the second phase, after ablation of the Nav1.8 population.

To summarise, we have developed AAV-GluClαβ.Cre^ON^ as a novel chemogenetic tool and used it successfully to silence nociceptive afferents in vitro and in vivo. Selective silencing of Nav1.8 positive neurons resulted in hyposensitivity to noxious mechanical, noxious thermal stimuli, and chemical pain. These findings provide evidence that our approach is a powerful means to selectively modulate subpopulations of sensory neurons and their circuitry.

## Conflict of interest statement

D.L.B. has acted as a consultant on behalf of Oxford Innovation for Abide, Amgen, G Mitsubishi, Tanabe, GSK, TEVA, Biogen, Lilly, Orion, and Theranexus.

## Appendix A. Supplemental digital content

Supplemental digital content associated with this article can be found online at http://links.lww.com/PAIN/B868.
