# Supplementary material for: GluCl.CreON enables selective inhibition of molecularly defined pain circuits
Source: Pain. 2023 Jun 27;164(12):2780–91. doi: 10.1097/j.pain.0000000000002976 (PMC10652717; doi:10.1097/j.pain.0000000000002976)
Supplement: Supplementary file 1 [file jop-164-2780-s001.pdf]

GluCl.Cre^{ON} enables selective inhibition of molecularly defined pain circuits

Steven J Middleton[1*], Huimin Hu[1], Jimena Perez-Sanchez[1], Sana Zuberi[1], Joseph McGrath Williams[1], Greg A Weir[2], David L Bennett[1].

1. Nuffield Department of Clinical Neurosciences, University of Oxford, Oxford, OX3 9DU, UK
2. School of Psychology and Neuroscience, College of Medical, Veterinary and Life Sciences, University of Glasgow, Glasgow, G12 8QQ, UK

Supplemental digital content: 3 Figures

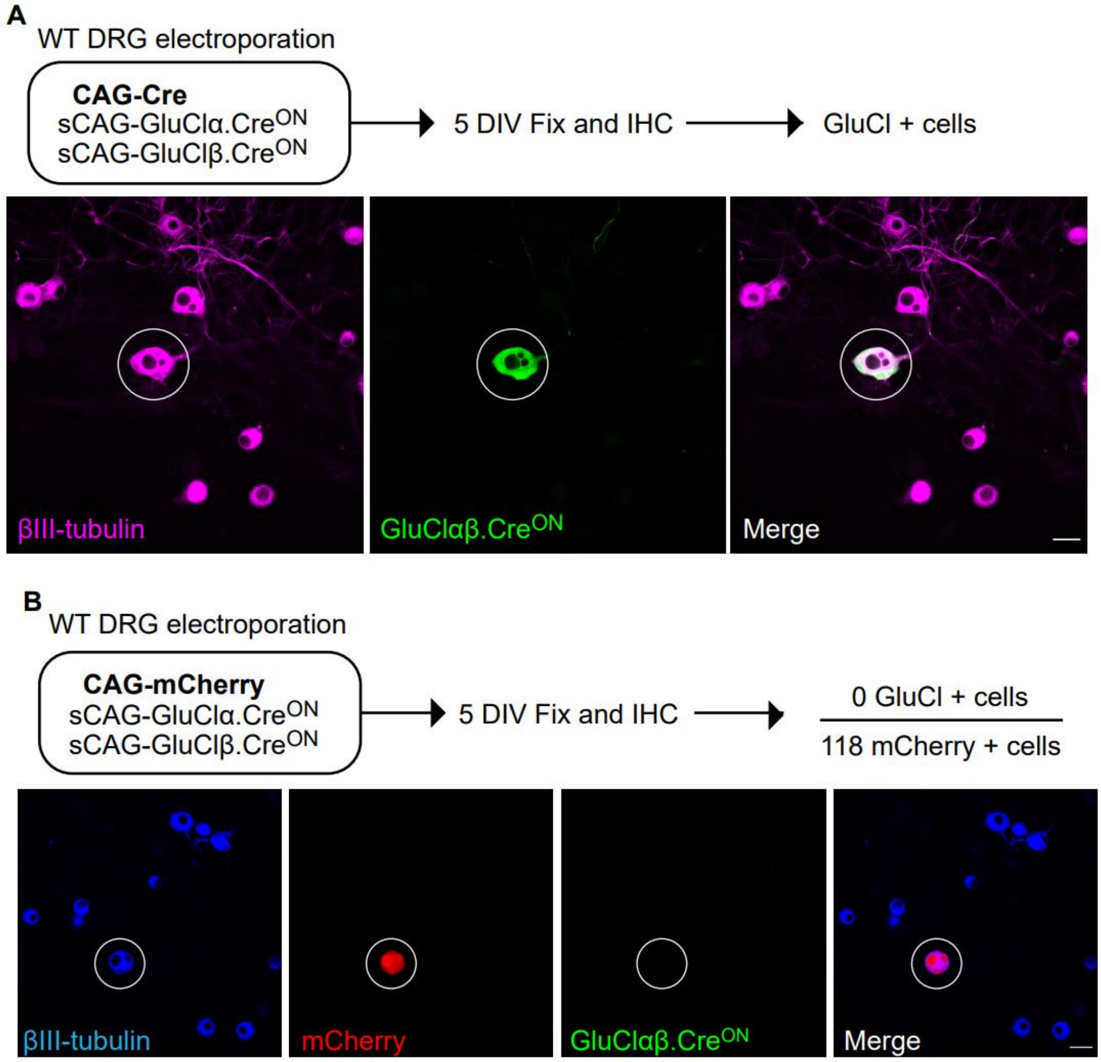

Supplemental Figure 1: GluClαβ.Cre^{ON} expression is selective for cre-recombinase *in vitro.* **A)** **E**xample image of a GluClαβ.Cre^{ON} positive neuron when co-transfected with cre-recombinase. **B)** WT DRG neurons co-transfected with mCherry instead of cre-recombinase. 118 mCherry+ neurons were counted (electroporated cultures from 3 mice), 0 were identified as being GluCl +. Scale bars, 25 µm.

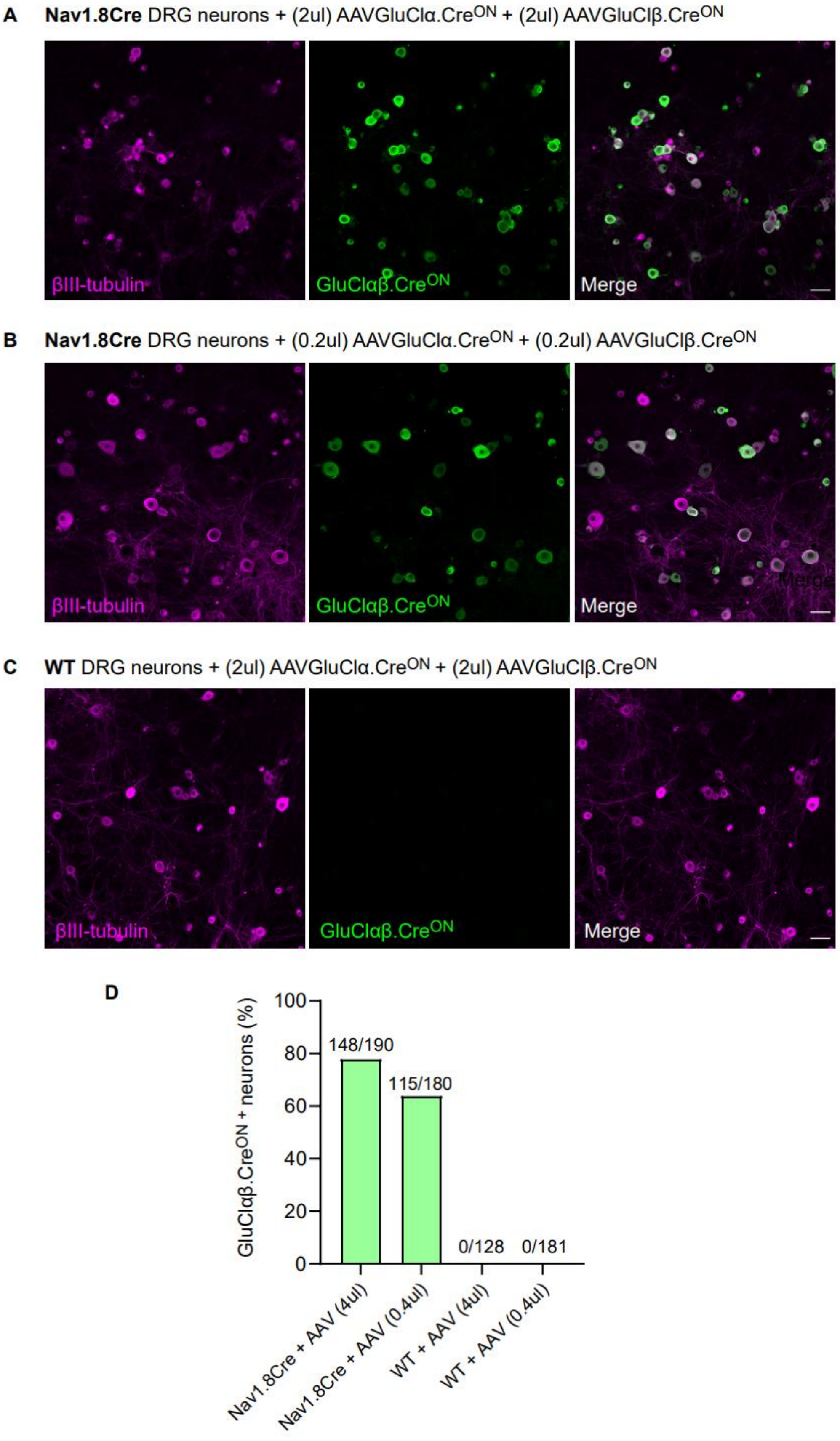
A
Nav1.8Cre DRG neurons + (2ul) AAVGluClα.Cre^ON + (2ul) AAVGluClβ.Cre^ON
βIII-tubulin
GluClαβ.Cre^ON
Merge
B
Nav1.8Cre DRG neurons + (0.2ul) AAVGluClα.Cre^ON + (0.2ul) AAVGluClβ.Cre^ON
βIII-tubulin
GluClαβ.Cre^ON
Merge
C
WT DRG neurons + (2ul) AAVGluClα.Cre^ON + (2ul) AAVGluClβ.Cre^ON
βIII-tubulin
GluClαβ.Cre^ON
Merge
D
GluClαβ.Cre^ON + neurons (%)
100
80
60
40
20
0
148/190
115/180
0/128
0/181
Nav1.8Cre + AAV (4ul)
Nav1.8Cre + AAV (0.4ul)
WT + AAV (4ul)
WT + AAV (0.4ul)

Supplemental Figure 2: AAV-GluClαβ.Cre^{ON} effectively transduces Nav1.8^{Cre} sensory neuron cultures but not wild type cultures. Example images of Nav1.8^{Cre} sensory neurons cultures receiving 2 (A) or 0.2 ul (B) of each AAV GluCl subunit. C) Example image of wildtype sensory neurons cultures receiving 2ul of each AAV subunit. D) Quantification of GluClαβ.Cre^{ON} positive neurons in each condition. GluClαβ.Cre^{ON} expression was absent in wild type cultures. (neuronal cultures from 3 mice per genotype). Scale bars, 50 µm.

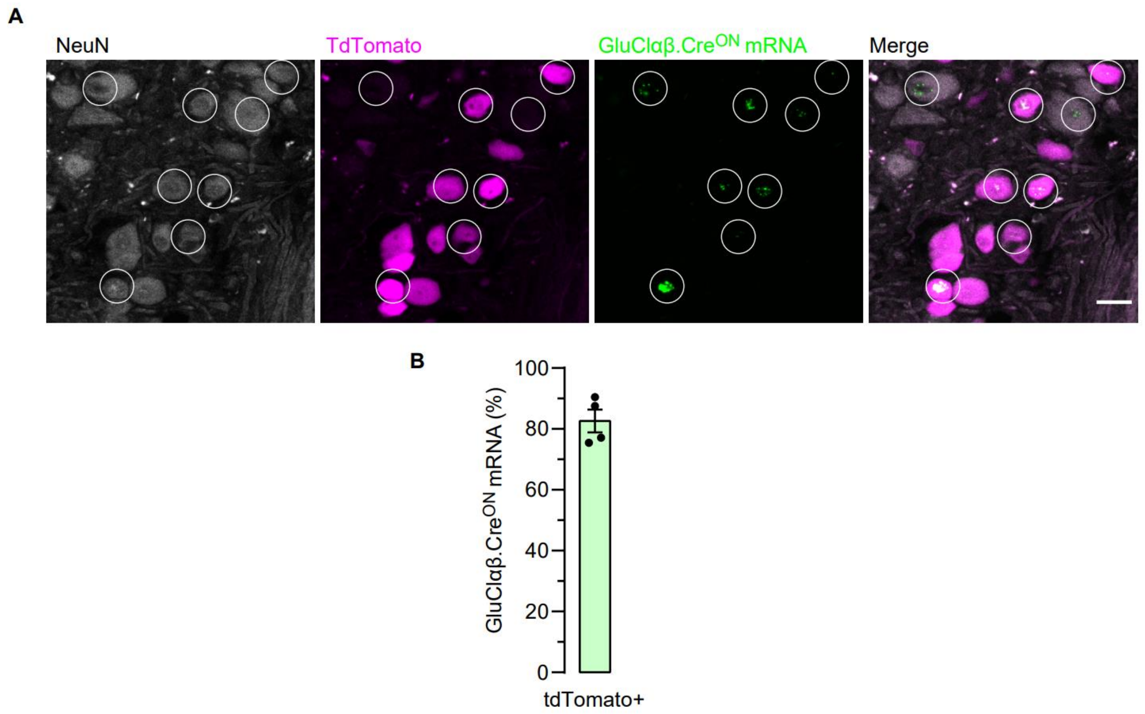

Supplemental Figure 3: AAV-GluClαβ.Cre^{ON} recombination specificity *in vivo*. A) Nav1.8^{Cre}tdTomato reporter mice were i.t. inject with AAV-GluClαβ.Cre^{ON} and DRGs harvested and analysed at least 6 weeks later. **B**) GluClαβ.Cre^{ON} mRNA positive neurons were detected and >80% co-localised with tdTomato. Scale bar 25 µm.
